# Use of indocyanine green angiography in microsurgical subinguinal varicocelectomy - lessons learned from our initial experience

**DOI:** 10.1590/S1677-5538.IBJU.2017.0107

**Published:** 2017

**Authors:** Chak-Lam Cho, Kwan-Lun Ho, Wayne Kwun-Wai Chan, Ringo Wing-Hong Chu, In-Chak Law

**Affiliations:** 1 Division of Urology, Department of Surgery, Kwong Wah Hospital, Hong Kong

**Keywords:** Varicocele, Microsurgery, Indocyanine Green

## Abstract

Microsurgical subinguinal varicocelectomy (MSV) is generally considered the gold standard nowadays in view of the lower risk of complications and recurrence. To achieve complete ligation of veins while preserving testicular artery (TA) during the procedure remains challenging despite the application of high power optical magnification and micro-Doppler ultrasonography. The use of intraoperative indocyanine green angiography (ICGA) with infrared fluorescence operative micro-scope in MSV potentially lowers the incidence of TA injury and shortens the learning curve of novice surgeons. We present our initial experience in the application of the technique in nine patients and explore the potential of the new adjunct.

## INTRODUCTION

Subinguinal microsurgical varicocelectomy (MSV) became the gold standard technique for varicocelectomy nowadays in view of the lower rate of recurrence and complications compared with open or laparoscopic techniques (1, 2). On one hand, subinguinal approach allows exposure of external spermatic and gubernacular veins and the lack of fascial incision results in less pain postoperatively. On the other hand, the more difficult dissection with a greater number of internal spermatic arteries and veins subinguinally (3) poses challenges to the operating surgeons. Injury to the testicular artery (TA) is a major complication of the procedure and a potential cause of testicular atrophy, but the incidence is unclear (4). It is believed that accidental arterial injury may go unnoticed and underreported particularly in non-microscopic varicocelectomy with inadequate optical magnification (5). Inspection of the cord for presence of arterial pulsations under high power magnification with irrigation of papaverine solution and the use of micro-Doppler (6, 7) are the most commonly adopted and effective means to locate the testicular artery (TA). However, the techniques require a certain level of experience and the result can be operator dependent. The application of indocyanine green angiography (ICGA) in MSV has been recently reported in the literature (8). The objective images provided by ICGA potentially simplify TA localization and decrease the incidence of inadvertent TA injury.

### Surgical Technique

Between September 2016 and January 2017, nine patients had unilateral MSV and ICGA performed on left grade 2 to 3 varicoceles in our unit. Four of the nine patients suffered from infertility with abnormal semen parameters. Two patients who presented with incidental finding of grade 3 left varicocele and oligozoospermia preferred surgical intervention after counseling. One patient was operated on due to bothersome discomfort associated with left grade 3 varicocele. Two varicocelectomies in adolescents were performed in view of testicular size discrepancy.

The procedures were performed under general anaesthesia. The infrared fluorescence operative microscope (Zeiss OPMI Pentero 900, Oberkochen, Germany) was brought into the field after incision of skin and spermatic fasciae. The vas deferens and its vessels were protected. ICGA was performed when the possible TA was identified. A pack of 25mg of indocyanine green (ICG) (Diagnogreen, Tokyo, Japan) was dissolved in 10mL of water. Each angiography required 5mL (12.5mg) of ICG solution which was prepared and administered by the anaesthetist in a bolus via a peripheral line. The Infrared 800 mode of the microscope was activated and the fluorescence angiography was recorded and analyzed. ICGA was repeated if necessary and at the end of the procedure to confirm a successful TA preservation.

**Figure 1:**
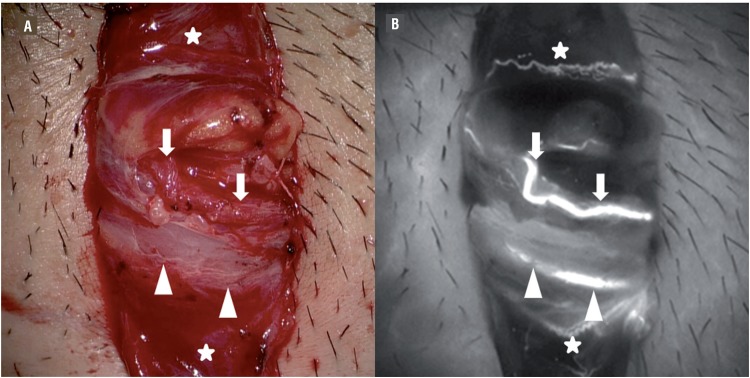
Intraoperative images during microsurgical subinguinal varicocelectomy. A) Microscopic view before injection of indocyanine green. B) Indocyanine green angiography clearly demonstrated all the arterial supply to the testicle. The testicular artery was marked by arrows. Deferential artery and cremasteric arteries were denoted by arrow heads and stars respectively.

**Figure 2:**
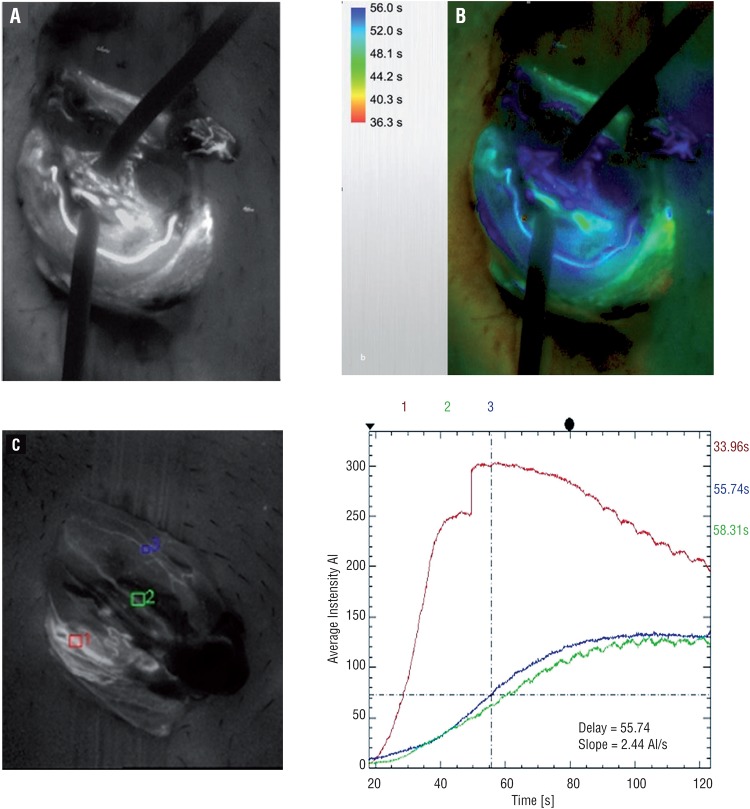
Built-in fluorescence modules of the operating microscope provided analysis of the vascular dynamics. A) Infrared 800 module demonstrates the relative intensity of indocyanine green signal. B) Flow 800 module illustrates the sequences the flow dynamics into a visual map. C) Interpretation of specific area on the angiographic image can be marked and D) Flow dynamic of each region can be illustrated in the form of curves.

Testicular artery was clearly identified by ICGA in all patients (Figure-1). Two testicular arteries were visualized in one patient while a single TA was identified in the remaining eight cases. The maximal diameter of the TA identified was no more than 1mm. All TA were shown up within one minute upon injection of ICG with a mean time of 36.3 seconds. Cremasteric and deferential arteries were visualized during intraoperative ICGA in most of the patients (Figure-1). The real-time angiographic images could be recorded and analyzed with the assistance of the built-in computer program of the operating microscope (Figure-2). The data could be presented in different formats by comparing the relative intensity and time to visualization of each vessel.

All patients were discharged the same day after the operation. No adverse reaction was observed after injection of ICG. No clinical recurrence and complication was recorded upon follow-up at 4 weeks after the operation.

## COMMENTS

**Figure 3:**
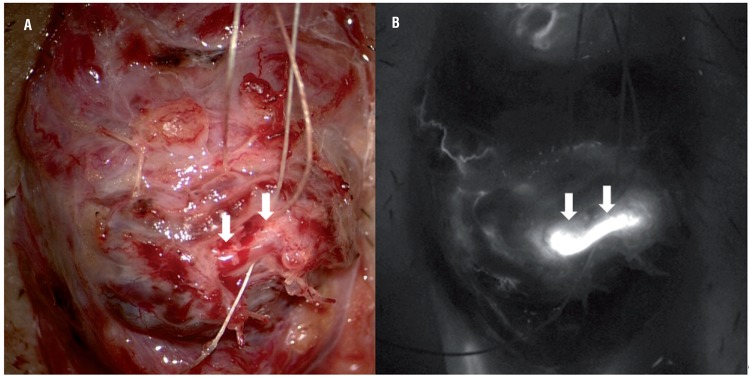
Intraoperative indocyanine green angiography may facilitate early identification of testicular artery. A) Microscopic view showing dense adhesions among intermingled artery and dilated veins which render the identification of arterial pulsation extremely difficulty. B) Indocyanine green angiography showed a single testicular artery among the densely adhered vessels.

The use of ICGA as an adjunct to MSV seems a promising technique in our initial experience. Localization of TA was achieved in all patients. The technique repeatedly demonstrated its ability in clearly identifying small TA of less than 1mm diameter. It was applicable to both adults and adolescents. ICGA is unique in providing an objective real-time assessment and images of arterial flow in the cord compared to direct visualization of pulsation under high power magnification and micro-Doppler. The intraoperative pictures can be recorded and are particularly useful for training and documentation purposes. It may facilitate transfer of technique to training surgeons and potentially shorten the learning curve. The technique of ICGA is not operator dependent and easy to administer with minimal prior preparation. Each ICGA spent no more than a few minutes and did not significantly prolong the operating time. The high-contrast images provided by the angiography allow simple interpretation to most surgeons. ICG has low toxicity with LD_50_ of 50-80mg/kg in animals (9). Confinement to the vascular compartment through binding with plasma proteins and rapid excretion via bile explained the safe nature of ICG. The safety (10) and short plasma half-life of ICG allows repeated ICG administration without compromising the quality of images. Its use was particularly valuable in patients with dense adhesion among intermingled arteries and veins (Figure-3). The adhesion rendered the identification of TA difficult by damping the arterial pulsation. The pulsation may appear weak and the exact localization of a particular pulsating artery may be difficult before the vessels were freely separated. ICGA may be superior in this scenario since the arterial flow is not obscured by adhesion among vessels. Small TA could be visualized before the adhesion was completely lysed. The whole course of TA across the operating field was shown up clearly most of the time. An earlier and more precise identification of TA during the procedure will reduce the risk of inadvertent arterial injury. Further comparative studies among the different strategies in TA preservation is required in delineating the potential advantages of ICGA in facilitating earlier TA identification and/or decreasing the risk of TA injury.

The recent advancement in fluorescence angiography lays in the analysis of ICG fluorescence dynamics. The built-in computer modules of the operating microscope provide data of flow dynamics of each vessel in the operating field. Although the significance of relative flow among testicular/deferential/cremasteric arteries in testicular blood supply is unknown, the demonstration of an intact collateral flow may be of importance in case of TA injury. An intact deferential and cremasteric supply may predict less probability of testicular atrophy and impairment of spermatogenesis after TA injury. In addition, ICGA may have a role in TA repair in case of accidental injury by localizing the abdominal end of the transected artery. The confirmation of intact deferential artery is preferred in the presence of prior groin or scrotal surgery when the status of the collateral supply is doubtful. The assessment of collateral arterial supply to the testes is not feasible with the technique of optical magnification and micro-Doppler.

The vascular anatomy and ICG dynamics illustrated by ICGA could be a research tool in better understanding the intraoperative microanatomy and physiology of varicocele. The information of microanatomy may further refine and decrease the complication of varicocelectomy.

ICGA may prove to be a more cost effective than the use of other adjunct such as micro-Doppler. Although the set-up of an infrared fluorescence operative microscope is more costly compared to a micro-Doppler machine (USD $283.000 versus $11.600), the microscope can be shared among different specialties in the setting of a multi-disciplinary hospital. The running cost of ICGA is much lower than micro-Doppler for each procedure. A pack of 25mg Diagnogreen costs around USD $43 in our locality and usually one to two packs were consumed for each procedure while a disposable micro-Doppler probe costs USD $386.

In conclusion, the use of intraoperative ICGA is safe and consistently provides objective assessment of testicular artery. The technique facilitates early identification and preservation of TA, and may decrease the incidence of TA injury during MSV. ICGA is potentially superior to and provides additional information compared to the current technique of TA identification with direct visualization of pulsation under high power magnification and micro-Doppler.
